# Gaia: segmented germanium detector for high-energy X-ray fluorescence and spectroscopic imaging

**DOI:** 10.1107/S1600577525009294

**Published:** 2026-01-01

**Authors:** Abdul K. Rumaiz, Francesca Capocasa, Anthony J. Kuczewski, Giovanni Pinaroli, Ji Li, John Kuczewski, Kristina Finnelli, Katherine Koh, Lorianne Shutlz-Johnson, Carter Fitzgerald, Thomas Krings, Nghia T. Vo, Michael Drakopoulos, Zhong Zhong, Thomas Caswell, D. Peter Siddons

**Affiliations:** a NSLS II Brookhaven National Laboratory Upton NY11973 USA; b Instrumentation Department Brookhaven National Laboratory Upton NY11973 USA; c Savannah River National Laboratory Aiken SC29808 USA; d Forschungszentrum GmbH, 52425Jülich, Germany; University of Malaga, Spain

**Keywords:** germanium detectors, high energy X-rays, fluorescence imaging, detectors

## Abstract

We present Gaia, a monolithic array of 96 high-purity germanium pixel detectors integrated with a custom low-noise application-specific integrated circuit (ASIC) and a field-programmable gate array (FPGA)-based data acquisition system. The sensor operates at ∼100 K using a commercial closed-cycle cryocooler, with the in-vacuum electronics thermally isolated from the cold finger to ensure thermal stability. The system demonstrates an average energy resolution of 711 eV at 122 keV, measured using a ^57^Co source, and 253 eV at 5.89 keV, measured with ^55^Fe across all channels. The readout architecture incorporates a high-performance FPGA paired with a dual-core ARM processor, forming a complete embedded Linux-based computing platform. Communication between the processor and FPGA is handled via memory-mapped I/O, and data are streamed over high-speed gigabit Ethernet. A full-scale 384-pixel Gaia detector, based on this 96-element module, is currently under fabrication.

## Abstract 2

We present Gaia, a cryogenically cooled monolithic array of 96 high-purity germanium pixel detectors integrated with a custom low-noise application-specific integrated circuit and a field-programmable gate array-based data acquisition system. The system achieves an average energy resolution of 711 eV at 122 keV and 253 eV at 5.89 keV, and serves as the foundation for a forthcoming 384-pixel detector.

## 1. Introduction

X-ray fluorescence (XRF) spectroscopy is a widely used technique for elemental identification and trace analysis across diverse scientific domains, including environmental monitoring, materials science and nuclear forensics (Sala *et al.*, 2025 ▸; Ryan *et al.*, 2014 ▸; Kopittke *et al.*, 2018 ▸). In particular, XRF offers a non-destructive approach to detect and characterize trace particles collected from environmental swipe samples. However, the current generation of laboratory-based and synchrotron XRF systems typically rely on silicon-based detectors operating in the 5–20 keV range. For high-*Z* elements, detection is predominantly limited to *L*-line emissions. This constraint introduces significant spectral overlap and ambiguity, particularly for high-*Z* elements, whose *L*-line emissions frequently coincide with *K* lines of common transition metals. These limitations can be addressed with a high-energy XRF platform that uses *K*-line fluorescence from actinide elements, providing enhanced sensitivity and spectral specificity (Meddouh *et al.*, 2023 ▸). Furthermore, *K* lines offer significantly higher fluorescence yields and cross sections than *L* lines, improving both the detection limit and accuracy of elemental identification in complex matrices. However, successful implementation of a *K*-line detection system requires a sensor capable of delivering high quantum efficiency and excellent energy resolution above 50 keV – capabilities beyond the reach of conventional silicon-based detectors.

High-purity germanium (HPGe) detectors are the gold standard for high-resolution X-ray spectroscopy, particularly in the hard X-ray regime. Germanium’s higher atomic number improves stopping power at elevated photon energies, while its intrinsically low Fano factor and well characterized charge transport properties enable superior energy resolution (Rumaiz *et al.*, 2018 ▸; Rumaiz *et al.*, 2014 ▸). Here, we present the development of Gaia, a monolithic pixelated germanium detector designed for high-flux high-energy XRF imaging applications. Gaia will feature an array of 384 cryogenically cooled pixels interfaced with a low-noise custom application-specific integrated circuit (ASIC) (Vernon *et al.*, 2020 ▸) and a field-programmable gate array (FPGA)-based readout system. The sensor operates near 100 K, stabilized by a commercial closed-cycle cryocooler, with the in-vacuum readout electronics thermally isolated from the cold finger to maintain low thermal load and system stability. To enhance further the utility of Gaia in real-world sample analysis, we plan to implement a fly-scan data acquisition approach using event-based triggering, which allows for continuous raster scanning of samples with minimal dwell time per pixel. This method significantly reduces overall scan duration and enables rapid high-resolution elemental mapping over large areas (Ryan *et al.*, 2010 ▸).

The prototype with 96 elements (mini-Gaia or 96-Gaia) has been validated using the High-Energy Engineering X-ray (HEX) beamline at the National Synchrotron Light Source II (NSLS-II), which provides a tunable high-brilliance X-ray source in the relevant energy range for actinide *K*-line excitation. The combination of high detection efficiency, excellent energy resolution and rapid data acquisition positions Gaia as a powerful new platform for high-energy micro-XRF, with immediate relevance to nuclear forensic science, catalysis, photovoltaics, electrochemical energy storage, nuclear waste management, high-performance coatings and emerging quantum materials.

## 2. Gaia sensor

**Figure 1:**
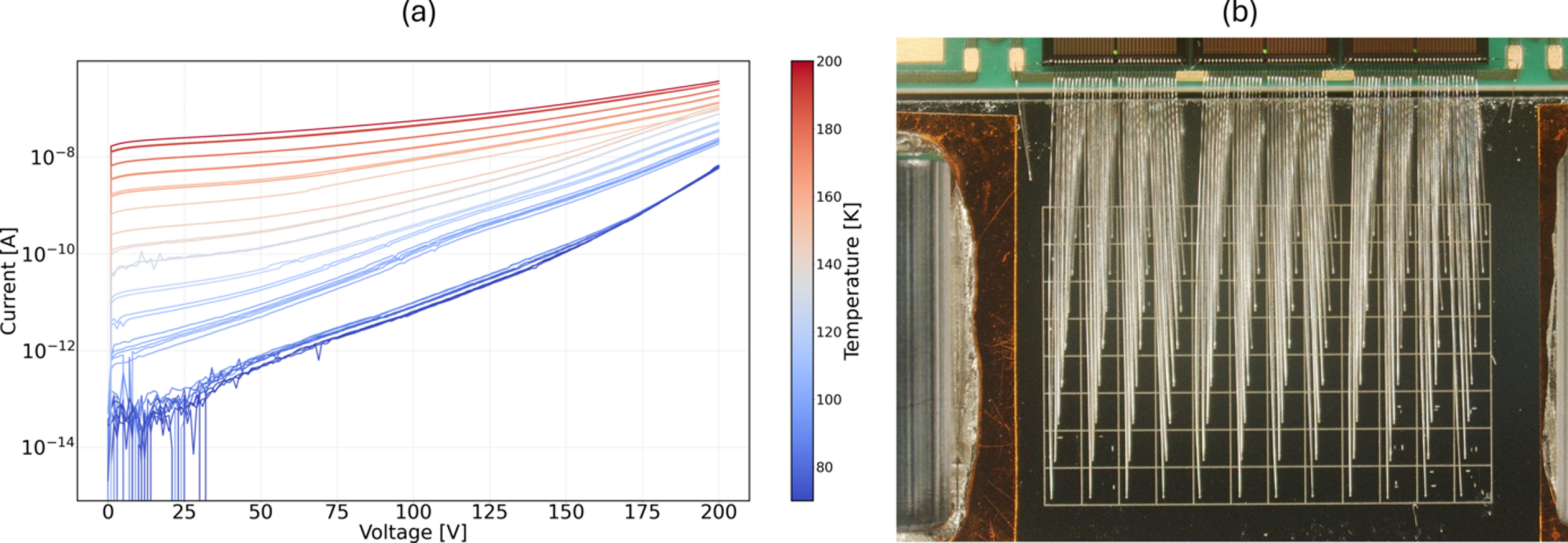
(*a*) *I*–*V* characteristics of the edge pixel at different temperatures. (*b*) The 96-detector mini-Gaia sensor wire-bonded to the HE-MARS readout ASIC.

All sensors used in the detector were fabricated using a well established fabrication process based on trench isolation to ensure effective electrical separation between pixels (Protic & Riepe, 1985 ▸). Briefly, a planar *p*–*n* junction is formed by uniformly implanting boron (B) ions on one surface to create a thin *p*-type layer, while a lithium (Li) diffused contact is formed on the opposite side to create a hole-blocking *n*-type region. Aluminium (Al), selected for its excellent electrical conductivity and adhesion, is deposited on both surfaces of the wafer by conventional magnetron sputtering to a thickness of approximately 200 nm. The Al metal electrode on the *p*-type side of the wafer is then segmented into the desired geometry by photolithography. Electrical isolation between adjacent pixels is then achieved by etching trenches using the Al layer as a mask. This approach offers a simple yet robust method for achieving high device yield and good pixel isolation. Prior to integration, the sensors were characterized in a cryogenic probe station to assess performance. Fig. 1 ▸(*a*) presents the current–voltage (*I*–*V*) characteristics measured at the edge pixel of the 96-Gaia sensor at different temperatures. An optical micrograph of a representative 96-Gaia wire-bonded to the readout ASIC is shown in Fig. 1 ▸(*b*). The 96-Gaia sensor has an active area of about 12 mm × 8 mm with a pixel pitch of 1 mm and a thickness of about 3 mm. For the measurement reported in this work, the Li side of the detector was in thermal contact with the cold finger for cooling. The detector was illuminated from the pixel side.

## 3. Gaia readout electronics

### 3.1. HE-MARS readout ASIC

The high-energy multi-element amplifier and readout system (HE-MARS) ASIC consists of 32 identical channels, each featuring a charge-sensitive preamplifier that can process either electron or hole signals. It offers four programmable gain settings, ranging from 1472 mV fC^−1^ to 184 mV fC^−1^ (corresponding to a full-scale photon energy of 25 keV up to 200 keV), and a shaping amplifier with four selectable shaping times from 0.25 µs to 2 µs (Vernon *et al.*, 2020 ▸). Each channel also incorporates a peak detector and a time-to-analog converter. The peak detector (De Geronimo *et al.*, 2002 ▸) captures photon energy and temporarily stores the value until it is retrieved by the readout system. Details of the HE-MARS ASIC, including noise performance, linearity and count-rate capabilities, can be found elswhere (Vernon *et al.*, 2020 ▸).

The timing system can operate in two modes: it can measure the length of time a shaped pulse remains above the channel threshold (used in systems to monitor pulse pile up) or it can measure the photon time of arrival. Data from each ASIC channel are transmitted through two differential analog outputs (PDO and TDO) along with the digital channel address. Photon events are read sequentially by the data acquisition (DAQ) module, and each event record includes the analog peak and time of arrival (or time over threshold), the channel address and the system clock for the absolute time of the event. This combined timing information enables accurate reconstruction of photon arrival times and identification of charge-sharing events through time coincidence analysis between adjacent strips (Rumaiz *et al.*, 2018 ▸). However, it should be noted that this capability was not utilized in the results presented in this paper.

**Figure 2:**
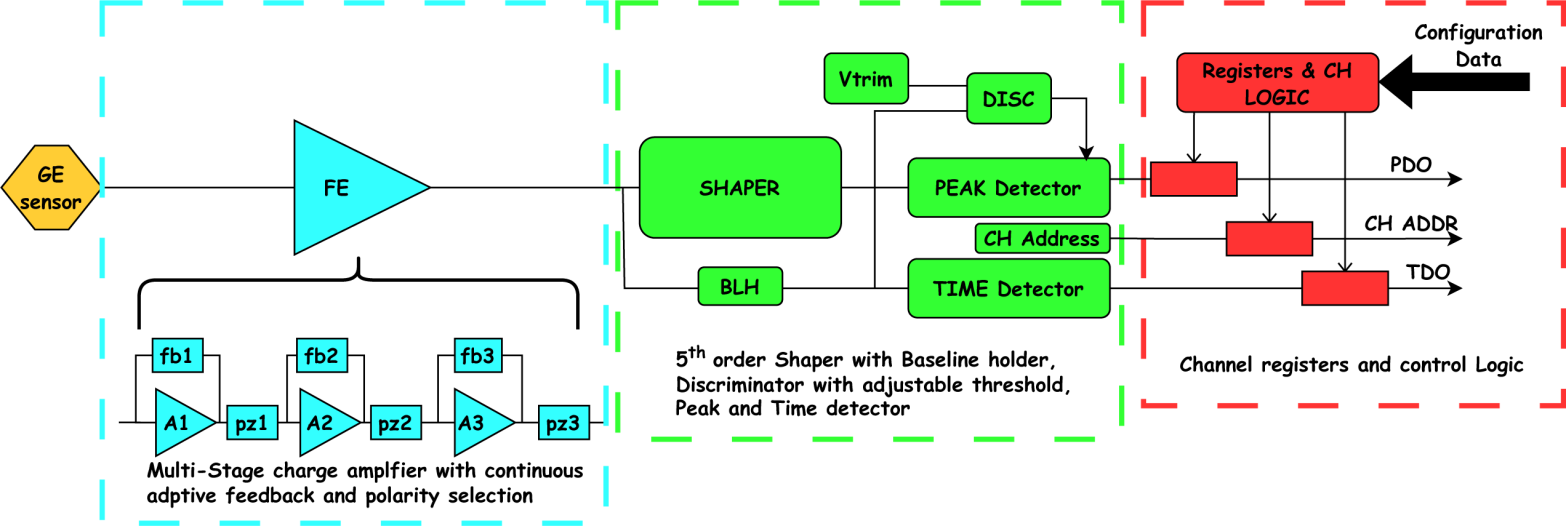
Architecture of a single HE-MARS channel.

Additionally, each channel includes a threshold discriminator, ensuring pulse processing only occurs when signals exceed this threshold. To correct for channel-to-channel variations caused by semiconductor process inconsistencies, the discriminator threshold includes both a global setting for the ASIC and a per-channel fine-tuning adjustment. This allows precise calibration and uniform performance across channels. Fig. 2 ▸ shows a block diagram of the ASIC front-end channel.

#### 3.1.1. The Ge readout module (GeRM)

**Figure 3:**
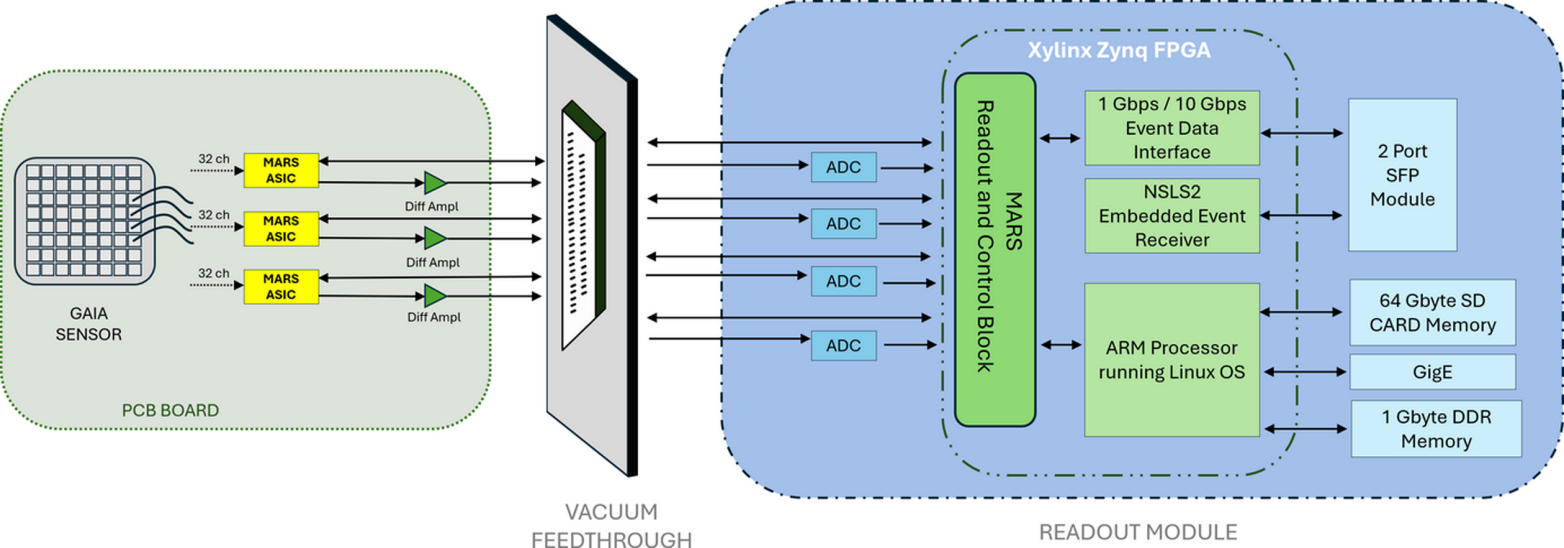
Block diagram of the complete detector system.

The interface module collects signals from up to three ASICs, digitizes them and transfers the data via Ethernet to a computer storage system. It consists of three main components: the in-cryostat printed circuit board (PCB), which houses the ASICs mounted near the sensor but is thermally decoupled from it; a vacuum-feedthrough assembly that routes approximately 90 signals through the vacuum enclosure to the processing electronics; and a processing readout module containing a high-performance FPGA with an embedded dual-core ARM processor, along with memory, storage, networking and other computing components. Fig. 3 ▸ shows a block diagram of the complete system.

The system is interconnected using a pair of rigid-flex PCBs, one located inside the vacuum and one in air. The readout module oversees the detector operation and runs a Linux operating system, allowing flexible and straightforward control software development. Linux communicates with the FPGA through memory-mapped ports corresponding to various detector control functions. This configuration delegates all real-time tasks to the FPGA while Linux handles slower functions, such as configuration and system monitoring.

Data transfer is handled via a standard gigabit Ethernet port, with additional high-speed connectivity provided by two SFP (small form factor pluggable) connectors for optional multi-gigabit fiber links. One SFP port is used to receive the NSLS-II event generator signal, synchronizing the detector’s data timestamps with the NSLS-II global clock. This design aligns with the NSLS-II data acquisition strategy, enabling precise timing and facilitating post-acquisition merging of multiple data streams.

## 4. Spectroscopy results

**Figure 4:**
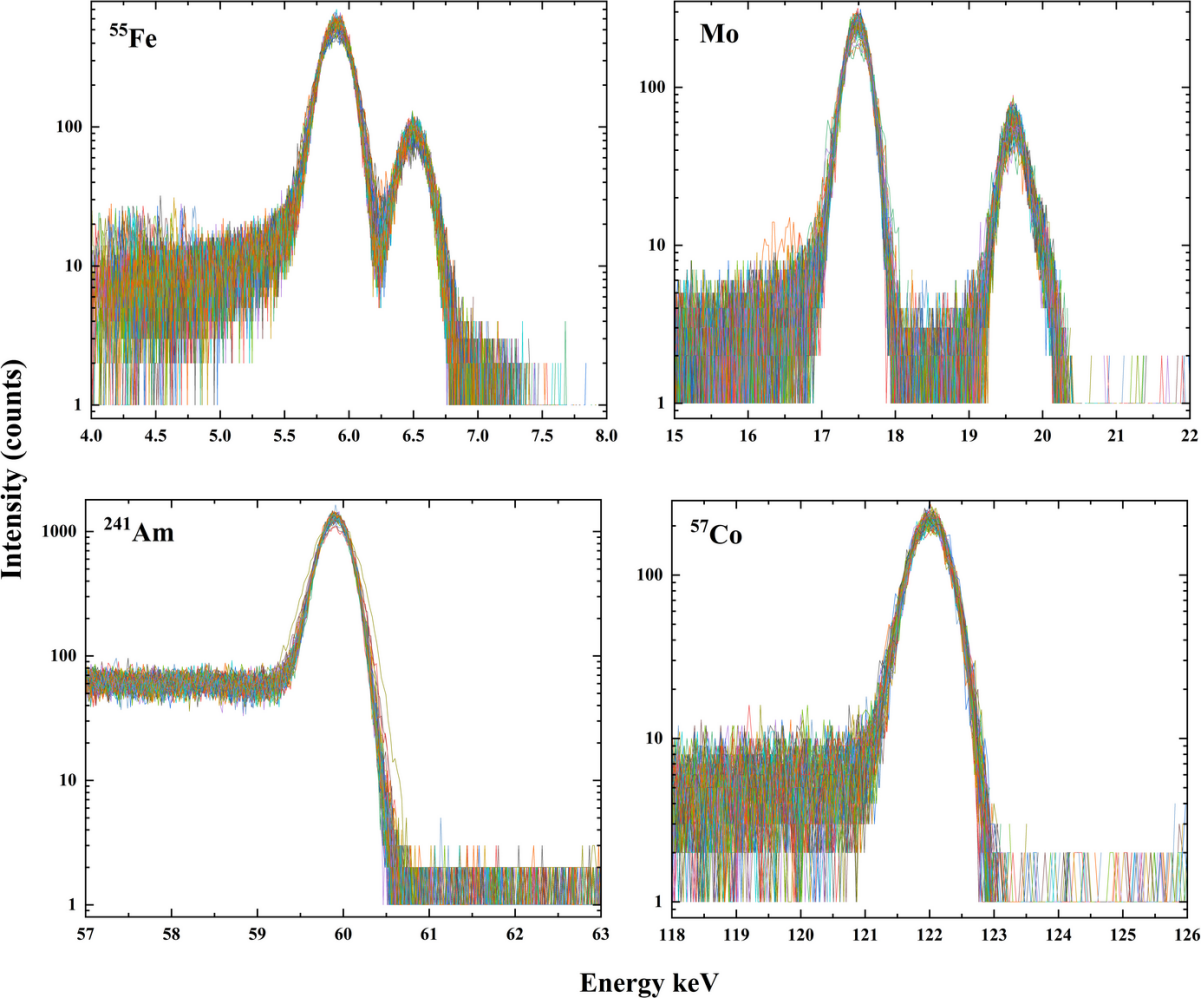
Response from ^55^Fe, *K*α emission from molybdenum (Mo), ^241^Am and a ^57^Co sealed source.

The germanium sensor is mounted on a copper cold plate, which is thermally connected to a Stirling-cycle cryocooler. This compact closed-cycle system maintains stable cryogenic temperatures near 100 K without the need for liquid cryogens. To minimize mechanical noise that could impact energy resolution, the cryocooler includes an active vibration damper on the cold head. A high voltage bias is applied to the unstructured lithium-diffused back contact. The detectors operate near liquid nitrogen temperatures, ensuring low leakage currents and stable performance. All reported data were acquired with a 1 µs peaking time which was found to provide the optimal noise performance. The performance of the detector was evaluated using sealed radioactive sources, including ^55^Fe, ^241^Am and ^57^Co, and *K*_α_ emission of molybdenum (Mo), to cover a broad spectral energy range. Fig. 4 ▸ displays the spectral response from all 96 channels, illustrating the detector’s consistency across the full 96-channel array. All the channels were found to be fully functional, demonstrating fairly uniform response and stable performance under irradiation.

It is well known that the energy resolution of a detector is fundamentally limited by the Fano factor, which accounts for statistical fluctuations in the number of charge carriers produced during photon absorption (Fano, 1947 ▸). This limit represents the best possible resolution achievable in an ideal system. However, in practical systems, the measured energy resolution is typically broader than the Fano limit due to several system-level noise sources, including electronic noise from the frontend ASIC and digitization electronics, and charge trapping and recombination within the detector bulk. Table 1 ▸ presents the measured energy resolutions at multiple photon energies for a representative channel, along with the corresponding Fano-limited values for direct comparison. Importantly, the detector demonstrates stable and high-resolution performance across a broad energy range spanning from tender X-rays (∼5 keV) to hard X-rays (up to 200 keV). As noted earlier, the detector was irradiated from the pixel side, where the effective dead region originates from the shallow *p*^+^ implantation and the 0.2 µm Al contact. Assuming an implantation depth of about 1 µm, the transmission remains above 75% at 5 keV. At the high-energy end, the quantum efficiency is instead limited by the detector thickness (3 mm Ge), resulting in an absorption of approximately 45% at 120 keV. This wide dynamic range highlights the detector’s versatility and robustness, enabling its use in a variety of applications requiring sensitivity to both low- and high-energy X-rays. The consistency of performance is further supported by a standard deviation of less than 5% across all 96 channels, indicating excellent response across the full pixel array.

The peak-to-background (P/B) ratio, defined as the ratio of the signal peak intensity to the average background level, is usually used to assess signal quality. In a standard ^55^Fe spectrum, the background is typically evaluated between 1.6 keV and 3.2 keV. However, in this measurement, a threshold setting was applied to the ASIC to suppress signals below 3.5 keV. Consequently, background was instead evaluated between 3.6 keV and 4.6 keV, yielding a P/B ratio of approximately 100:1. It should be noted that no collimator was used during this measurement and the use of an appropriate collimator is expected to improve the P/B ratio further.

## 5. High-energy fluorescence imaging on HEX

**Figure 5:**
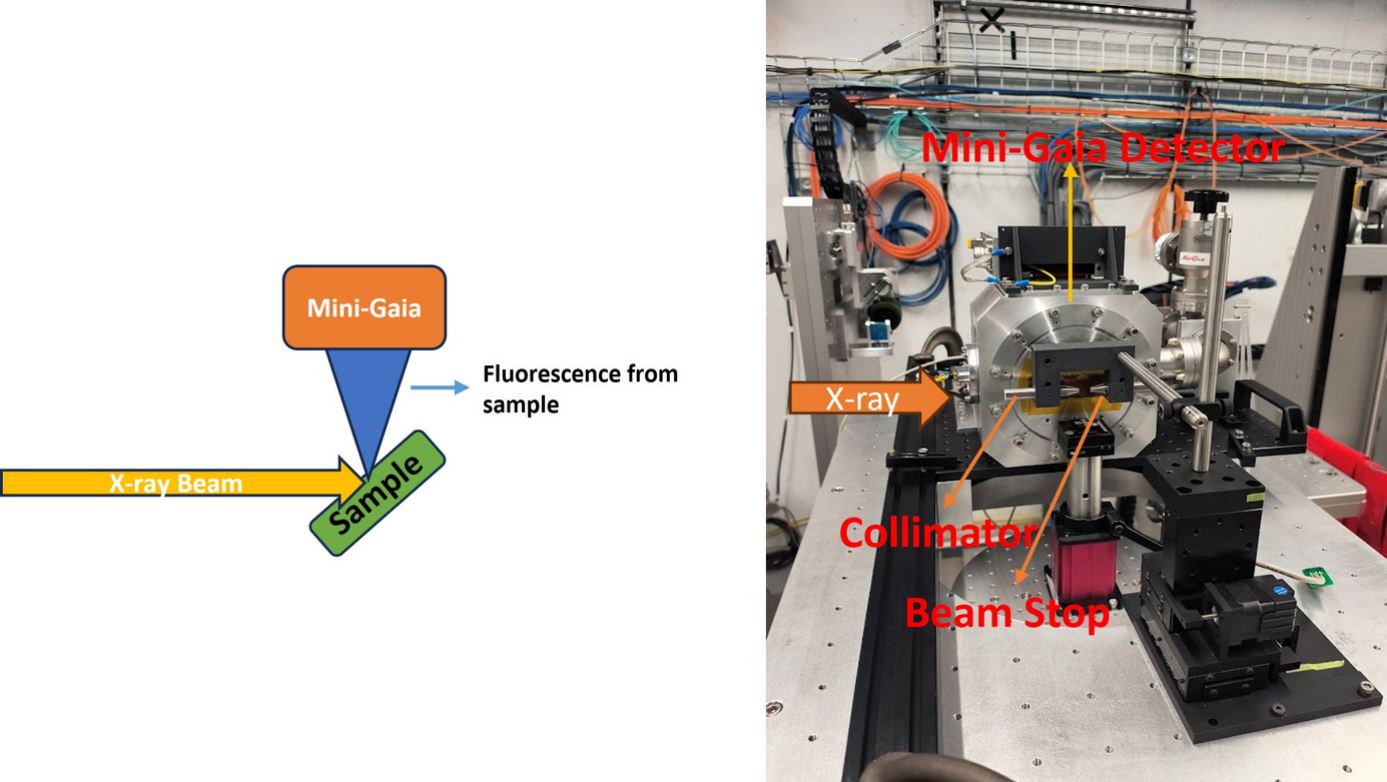
Measurement setup: (left) schematic diagram and (right) a setup photograph of the mini-Gaia detector on the HEX beamline.

High-resolution fluorescence spectra and images were collected on the HEX beamline endstation at the NSLS-II. Details of the beamline can be found elsewhere (Drakopoulos *et al.*, 2024 ▸; Zhong *et al.*, 2021 ▸). We performed the experiments with a monochromatic beam (above the uranium *K* lines) and with a white beam. Fig. 5 ▸ shows the experimental set up and a picture of the detector as installed on the HEX beamline.

**Figure 6:**
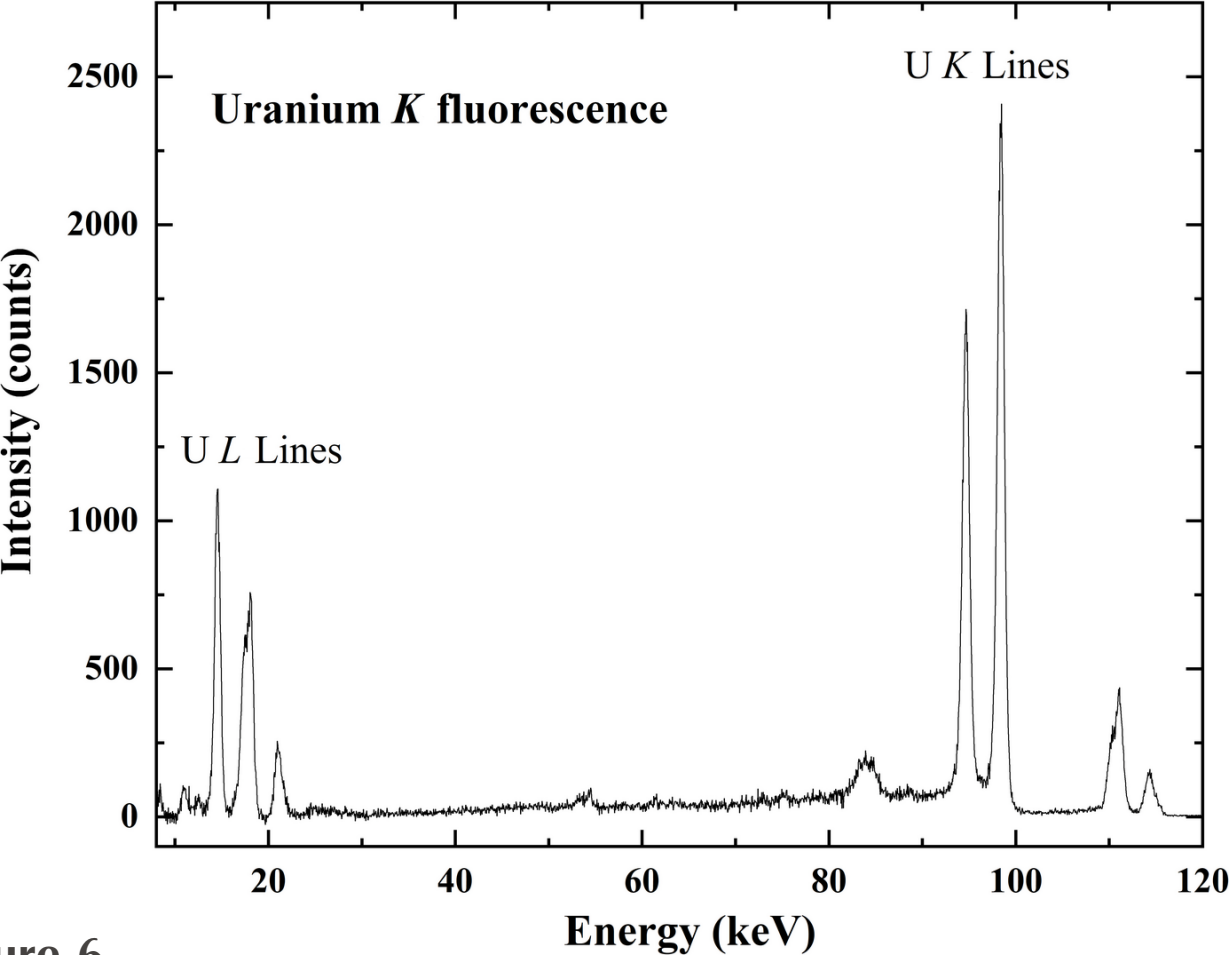
Full XRF spectrum of the reference U sample. Data collected at 122 keV.

Initial measurements were performed on a standard uranium (U) reference sample to obtain a clean U fluorescence spectrum which was used for calibration purposes. Fig. 6 ▸ shows the spectrum for the reference U sample. The spectrum shows the characteristic U *K*α_1_ and *K*α_2_ lines at 98.4 keV and 94.7 keV, respectively, along with the *K*β lines at 110.3 keV, 111.2 keV and 114.3 keV. Additionally, we also see the well separated low-energy *L* lines at around 20 keV. The weak peak close to 85 keV corresponds to the Compton scattering peak. It is worth noting that the thorium (Th) *K* lines are separated from the U *K* lines by about 5 keV and, given the detector’s energy resolution, these lines should be readily distinguishable. While Th *K* lines are not expected in the spectrum from a pure U source, this comparison highlights that the detector is capable of resolving neighboring actinide *K* lines, a distinction that is more challenging for their closely spaced *L* lines. This is particularly important for applications where both U and Th may be present.

Fluorescence data clearly show that for high-*Z* elements, including actinides, the *K* lines are generally preferred for fluorescence-based measurements due to their significantly higher fluorescence yield and well separated high-energy spectral features. The *K*-shell fluorescence yield in these heavy elements exceeds well over 95%, implying every *K*-shell ionization results in the emission of a characteristic X-ray. In contrast, *L*-shell fluorescence yields are notably lower (typically around 40–50%) (Hubbell *et al.*, 1994 ▸) due to relaxation occurring through non-radiative Auger processes.

**Figure 7:**
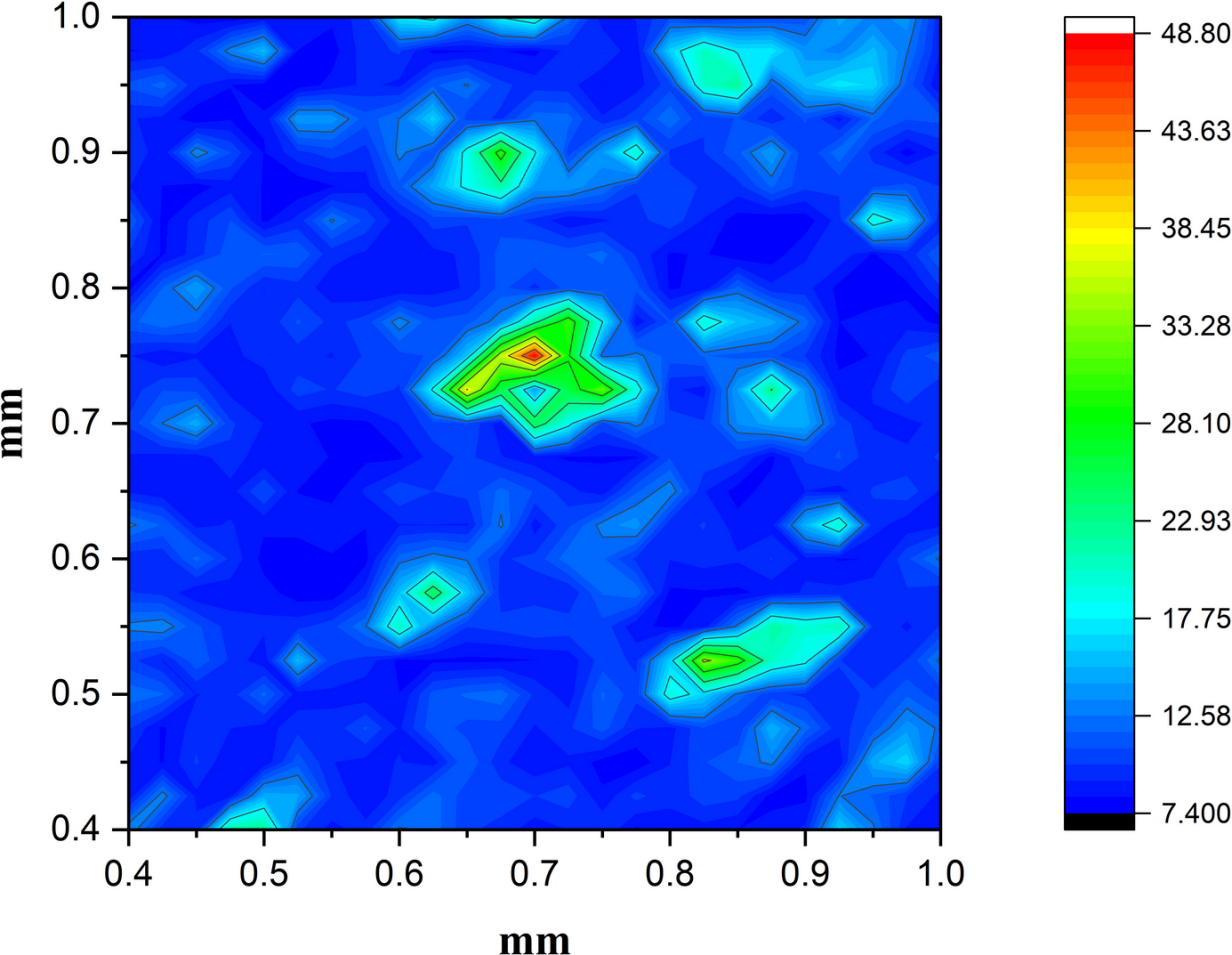
Spectroscopic fluorescence map of the uranium reference powder sample, generated by mapping the net intensity of the U *K*α_1_ line across a 1 mm × 1 mm area. The image was acquired using a 40 µm step size and a 50 µm white-beam spot.

To enable spectroscopic imaging of the U reference sample, we carried out a high-resolution fluorescence scan over a defined 1 mm × 1 mm region of the powder, which was securely sandwiched between layers of Kapton tape to hold it in place without introducing significant background signal. The step scan was performed with a step size of 40 µm and the incident X-ray beam was focused to a 50 µm spot using precision slits with a dwell time of 3 min per step. To enhance the photon flux and reduce the acquisition time, measurements were performed using a white (broadband) beam rather than a monochromatic source. Of particular concern during measurments was the overlap of the Compton scatter peak with the U *K* lines. To address this, we implemented a tungsten collimator and a strategically placed beam stop (as illustrated in Fig. 5 ▸) to suppress forward-scattered X-rays and reduce the Compton contribution to the measured spectra. The collected fluorescence spectra at each scan position were subsequently processed using user-developed Python routines, which performed energy calibration, peak fitting and background subtraction. The extracted net intensities of the U *K* lines were then used to construct high-resolution elemental maps, revealing the spatial distribution of uranium within the scanned region. Fig. 7 ▸ shows the resulting spectroscopic image, clearly revealing the spatial distribution of uranium within the scanned region.

## 6. Future work

We are currently developing a 384-channel version of the mini-Gaia detector. This upgraded design includes a central aperture in the sensor to allow the incident beam to pass through, enabling measurements in backscattering geometries (Siddons *et al.*, 2014 ▸). This configuration is expected to improve the effective solid angle significantly and enhance collection efficiency. In parallel, a new FPGA architecture is being designed to integrate detector data acquisition with the position feedback from the stepper motor encoder. With real-time spectral deconvolution implemented directly on the FPGA, this system will support continuous ‘fly-scan’ fluorescence imaging, eliminating the need for step-and-acquire acquisition and enabling faster high-throughput spectroscopic mapping.

## 7. Conclusions

In summary, the Gaia detector is a major step forward in high-energy XRF imaging by integrating a cryogenically cooled array of high-purity germanium detectors with custom low-noise ASICs and FPGA-based data acquisition. Operating at stable cryogenic temperatures near 100 K, Gaia achieves exceptional energy resolution across an expansive energy range, from tender to hard X-rays, surpassing the intrinsic limits of conventional silicon-based detectors. This capability allows for reliable detection of *K*-line fluorescence from high-*Z* elements, which provides significantly higher fluorescence yields and clearer spectral signatures than traditional *L*-line emissions.

The mini-Gaia prototype has been tested and validated with sealed sources and on the HEX beamline at NSLS-II. The detector produced high-resolution fluorescence spectra and detailed elemental maps from uranium samples.

These advancements hold immediate promise for critical applications in nuclear forensics, catalysis and quantum materials research, where precise elemental identification is paramount. Future efforts are focused on scaling up to a full 384-pixel Gaia detector featuring a central aperture to enhance collection efficiency, coupled with a redesigned FPGA architecture that will enable continuous fly-scan fluorescence imaging. This next generation will facilitate faster high-throughput mapping, paving the way for broader adoption and transformative impact across multiple scientific fields.

## Figures and Tables

**Table 1:** Measured full width at half-maximum (FWHM) of a representative channel

	Energy	Measured FWHM	Fano limit
^55^Fe	5.89 keV	252 ± 1 eV	111 eV
Mo *K*_α_	17.44 keV	360 ± 1 eV	191 eV
^241^Am	59.54 keV	406 ± 2 eV	353 eV
^57^Co	122 keV	697 ± 2 eV	504 eV

## Data Availability

The data supporting the research findings are not publicly accessible but can be obtained from the authors by contacting them directly.
